# High Salinity Reduces Plant Growth and Photosynthetic Performance but Enhances Certain Nutritional Quality of C_4_ Halophyte *Portulaca oleracea* L. Grown Hydroponically Under LED Lighting

**DOI:** 10.3389/fpls.2021.651341

**Published:** 2021-03-22

**Authors:** Jie He, Xueli You, Lin Qin

**Affiliations:** National Institute of Education, Nanyang Technological University, Singapore, Singapore

**Keywords:** dietary minerals, hydroponics, LED lighting, photosynthesis, phytochemicals, *Portulaca oleracea* L.

## Abstract

*Portulaca oleracea* L. (known as purslane) is one of the most nutritious leafy vegetables owing to its high content of antioxidants. In this study, all plants were grown indoors hydroponically with different NaCl salinities. Photosynthetic photo flux density (PPFD) at 200 μmol m^−2^ s^−1^ (12 h) was provided to all plants by LED with red:blue ratio of 2.2. Thirty days after transplanting, plants grown with100 mM NaCl had the highest productivity and the fastest leaf growth followed by those with 0, 200 and 300 mM NaCl. Grown with 300 mM NaCl, purslane had the lowest specific leaf area due to its highest leaf dry matter content and its lowest water content. All plants had similar values of leaf succulence except for those with 300 mM NaCl. Total chlorophyll and carotenoids contents were significantly higher in plants grown with 0 and 100 mM NaCl than with 200, and 300 mM NaCl. All plants had F_v_/F_m_ ratios close to 0.8. However, electron transport rate and ΔF/F_m_′ were significantly higher in plants grown with 0 and 100 mM NaCl than with 200 and 300 mM NaCl. CAM-induced purslane with 300 mM NaCl had higher non-photochemical quenching. Maximum net photosynthetic O_2_ evolution rate and Cyt *b_6_f* concentration were significantly lower with 300 mM NaCl compared to all other plants while all plants had similar PS II concentration. Proline concentration increased with increasing salinities. All plants had similar levels of total soluble sugars. Plants grown with 0 and 100 mM NaCl had significantly higher concentrations of NO_3_^−^, total reduced nitrogen, total leaf soluble protein, Rubisco protein, total ascorbic acid, and total phenolic compounds than with 200 and 300 mM NaCl. The highest concentrations of K, Ca, and Mg were found in purslane grown under 0 mM NaCl. Statistically, no significant differences in Fe concentrations were observed among all plants. However, salinity seems to increase Fe concentration. In conclusion, it is feasible to grow purslane under 100 mM NaCl as it is the most optimal condition to achieve higher productivity and better quality. However, the production of antioxidants may depend on not only salinity but also other growth conditions.

## Introduction

According to the International Water Management Institute, agriculture, which accounts for about 70% of global water withdrawals, is constantly competing with domestic, industrial, and environmental uses for a scarce water supply. Singapore’s biggest threat is the water crisis. An integrated approach, therefore, is imperative for increasing food security. To increase food supply locally, the utilization of seawater to produce halophytes as vegetables hydroponically, which uses much less water than traditional farming, could be a strategy to address water scarcity in Singapore. The almost infinite availability of seawater highlights the importance of halophytes as a source of vegetable crops, particularly since they do not compete with glycophytic food crops (Ventura et al., 2015).

*Portulaca oleracea* L. (known as purslane) is a nutritious leafy vegetable mainly native to the Mediterranean basin and widespread throughout the world (Karkanis and Petropoulos, 2017; Petropoulos et al., 2018). Purslane also has medicinal properties (Uddin et al., 2014; Petropoulos et al., 2016), and it has been listed among the most common used plants for medicinal purposes by WHO (Naeem and Khan, 2013). Interest in cultivating purslane as a vegetable has increased since its bioactive compounds, such as omega-3 fatty acid (Palaniswamy et al., 2001; Teixeira et al., 2010; Bessrour et al., 2018) and ascorbic acid, together with other antioxidants, vitamins, and essential amino acids have been identified (Franco et al., 2011). According to Yazici et al. (2007), purslane is a succulent dicot halophyte, which is drought- and salt-tolerant. However, Kafi and Rahimi (2011) reported that purslane is considered as a moderately salt tolerant species, with a capacity to withstand soil salinity only up to 240 mM. Cultivating purslane under the same NaCl salinity levels hydroponically, Anastaćio and Carvalho (2013) did not observe any toxicity symptoms. Moreover, it was reported that the salinity tolerance of purslane is significantly decreased when plants are grown under low light intensity (Franco et al., 2011). Apart from utilizing the seawater resources to mass produce halophyte vegetables, to compensate for the lack of available land, Singapore also needs to develop vertical farming systems to produce potential edible halophyte vegetables under LED lighting. Our recent studies also showed that an edible halophyte leafy vegetable, *Mesembryanthemum crystallinum* (ice plant) grown indoors was affected by NaCl salinity (He and Qin, 2020a) and induced drought stress (He et al., 2020) as well as LED spectral quality when plants grown with freshwater (He et al., 2017). These studies show that salinity, water supply, and light quality affect productivity, photosynthetic performance, and nutritional quality of *M. crystallinum* grown indoors. However, a very little research has been done on the effects of salinity on growth, photosynthetic performance, and nutritional quality of purslane grown indoors under LED lighting with constant levels of light intensity.

*Purslane* is the only genus known to have both C_4_ and crassulacean acid metabolism (CAM). It is also well-known CAM was elicited if *purslane* was subjected to drought stress (Koch and Kennedy, 1982; Lara et al., 2004; D’Andrea et al., 2014; Winter and Holtum, 2014; Mulry et al., 2015). Lara et al. (2004) reported that the succulent purslane plant shifted its C_4_ photosynthetic metabolism to CAM after 23 days of withholding water. In another study with purslane, by measuring the net CO_2_ exchange for the above-ground tissues of a young purslane, Winter and Holtum (2014) found that net CO_2_ uptake was observed in the dark but CO_2_ exchange in the light was limited to a short burst at the beginning of the light period after no watering for 10 days. However, the CO_2_ fluxes increased during the light and the dark, reverting to a non-CAM pattern within 24 h after re-watering. Ferrari et al. (2020) evaluated CAM plasticity of 11 subspecies of *P. oleracea* from distant geographical locations, and they concluded that all subspecies expressed CAM in a fully-reversible manner. However, there are different combinations of CAM expression level within the *P. oleracea* complex. When CAM was induced in purslane under drought stress, D’Andrea et al. (2014) found that chlorophyll fluorescence parameters were transitorily affected and non-radiative energy dissipation mechanisms were induced by measuring the effective quantum yield of PSII (ΔF/F_m_′), the photochemical quenching coefficient (qP) and non-photochemical quenching (qN or NPQ). In the study of halophyte *M. crystallinum* L., Broetto et al. (2007) found that, when CAM was induced in this species under combination of salinity and high light, the operation of photosystem II (PS II) was affected. The electron transport rate (ETR) and ΔF/F_m_′ were reduced while NPQ was increased (Broetto et al., 2007). Our recent studies on *M. crystallinum* L. also showed the similar results (unpublished). These results suggest that CAM induction and its relation to PS II photochemistry make it possible to evaluate photosynthetic performance and the extent of its tolerance to salinity stress. However, to the best of our knowledge, there is very little study on the upregulation of CAM in C_4_ purslane under salinity stress.

Accumulations of bioactive compounds have been investigated extensively in purslane (Zhou et al., 2015). When a CAM is induced under drought stress and then reversed back to C_4_ upon re-watering in purslane, there was a clear metabolic shift, implying that antioxidants may be involved in photosynthetic machinery protection. Increases in osmolytes, such as proline and total soluble sugar (TSS), could contribute to withstand drought (Rahdari et al., 2012; D’Andrea et al., 2014; Jin et al., 2016). Salt stress also results in high accumulation of proline in purslane (Yazici et al., 2007). Not only proline, but also TSS that can be utilized in functional food, are produced for protection against hyperosmotic stress (Flowers and Colmer, 2008; Agarie et al., 2009; Hsouna et al., 2020). Sdouga et al. (2019) highlighted a correlation between the gene expression varying by population and saline concentration and the level of proline in the leaves of *P. oleracea*. Apart from the accumulation of proline, halophytes are also able to synthesize natural antioxidants such as ascorbic acid and total phenolic compounds under saline and drought conditions (Dat et al., 2000; Ksouri et al., 2007; He et al., 2020). According to Uddin et al. (2012a), the antioxidant potential of purslane was indeed mainly dependent on the total phenolic content. However, Lim and Quah (2007) reported that there was a great variation in the accumulation of total phenolic compounds, which was directly dependent on the season of the year.

Dietary minerals also contribute to the valuable nutritional profile of purslane. Potassium (K), calcium (Ca), magnesium (Mg), and iron (Fe) are abundant in purslane plant (Uddin et al., 2012b). The effects of salinity on mineral composition of purslane leaves have been reported by a number of researchers. For instance, Teixeira and Carvalho (2009) concluded that high salinity resulted in decreases of Ca, K, and Zn contents. Significant differences of the mineral compositions have been also observed among different salinity levels (Uddin et al., 2012b). Chowdhar et al. (2013) reported that Ca was negatively correlated with Na, while there were positive correlations among Na and K, Mg, and Fe.

Impacts of drought stress have been studied frequently in purslane, and the induction of CAM could reduce the negative effects of drought stress. *Portulaca oleracea* engages multiple strategies to cope with the drought and CAM induction is a metabolic strategy of this species to maintain photosynthesis under drought stress (D’Andrea et al., 2014; Ferrari et al., 2020). However, there is little published work exploring the expression of CAM, growth, physiological responses, and nutritional quantity of purslane to salinity when they were grown hydroponically indoors under LED lighting. Thus, this study aimed to address the following objectives: (1) explore the application of saline water to grow purslane hydroponically under LED lighting by studying shoot and root productivity, leaf growth, and its water status; (2) study the impacts of salinity on photosynthetic performance of purslane including photosynthetic light use efficiency, photosynthetic O_2_ evolution, CAM acidity, and the concentrations of PS II and Cyt *b_6_f*; and (3) study nutritional quality including both phytochemical and dietary mineral accumulations.

## Materials and Methods

### Plant Materials and Culture Methods

**Figure 1:**
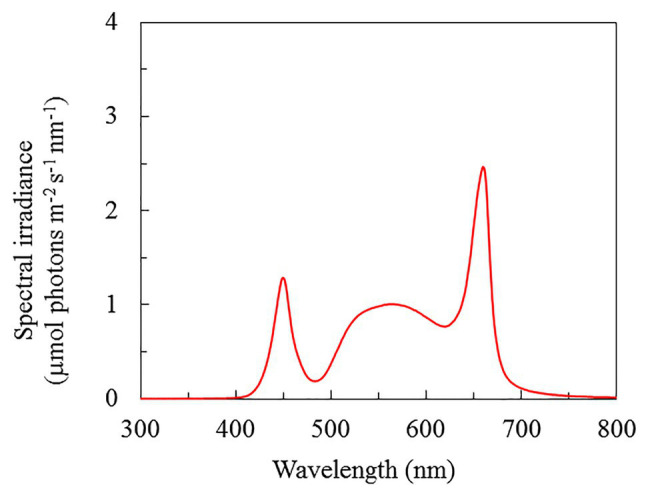
Light spectral distribution of *P. oleracea* grown hydroponically with different NaCl salinities. Spectral scans were recorded every 0.5 nm with a spectroradiometer (PS300, Apogee Instruments, United States).

Seeds of purslane (*P. oleracea* L. cv. POR – 2936) produced in Holland were germinated on filter papers before being inserted into polyurethane cubes. All seedlings were incubated under a photosynthetic photon flux density (PPFD) of 100 μmol m^−2^ s^−1^ provided by high-pressure sodium lamps for 5 weeks. Seedlings were then transplanted into an indoor hydroponic systems and were grown under red/blue LED ratios of 2.2 (WR-16 W, Beijing Lighting Valley Technology Co., Ltd., China), and the light spectral distribution is shown in Figure 1. All plants were exposed to the same level of PPFD of 200 μmol m^−2^ s^−1^, 12 h photoperiod and were grown under three NaCl salinities by adding 100, 200, and 300 mM NaCl, respectively, to a full-strength Netherlands Standard Composition with 2.2 ± 0.2 mS cm^−1^ conductivity and pH 6.0 ± 0.2. The room temperature and relative humidity were 24.5/23°C and 56/82% (day/night), respectively.

### Measurements of Shoot and Root Productivity, Leaf Growth, and Leaf Water Status

Plants from each treatment were harvested after 30 days of transplanting. Total leaf number was recorded. Shoot (leaves + stem) and root were separated for fresh weight (FW) measurement. Total leaf area (TLA) was measured using a leaf area meter (WinDIAS3 Image Analysis system). Shoot and roots were then dried at 80°C for 4 days, before re-weighing them to obtain dry weight (DW). Specific leaf area (SLA) was determined as L_a_/L_DW_ where L_a_ = leaf area (cm^2^) and L_DW_ = leaf dry weight (g; Hunt et al., 2002). Leaf succulence (LS) was estimated as L_FW_/L_a_ where L_FW_ = leaf FW (Agarie et al., 2007). Leaf dry matter content (LDMC) was determined by L_DW_/L_FW_ (Garnier et al., 2001). Leaf water content (LWC) was determined as (L_FW_−L_DW_)/L_FW_.

### Measurements of Total Chlorophyll and Carotenoids Concentration

Twenty-six days after transplanting, leaf discs of fully expanded young leaves were weighed and placed in 5 ml of N, N-dimethylformamide (N,N-DMF, Sigma Chemical Co.) in darkness for 48 h at 4°C. The absorption of pigments was measured using a spectrophotometer (UV-2550 Shimadzu, Japan) at 647, 664, and 480 nm, respectively. Chl a, Chl b and Car concentrations were calculated as described by Wellburn (1994).

### Measurement of Chl Fluorescence F_v_/F_m_ Ratio

Maximum quantum yield of PSII was estimated in dark-adapted attached fully expanded young leaves by the F_v_/F_m_ ratio during mid-photoperiod using the Plant Efficiency Analyzer (Hansatech Instruments, UK) according to He et al. (2011).

### Measurements of Electron Transport Rate, Effective Quantum Yield of PSII, and Non-photochemical Quenching

The whole young fully expanded leaves were placed on moist filter papers in Petri dishes. They were pre-darkened for 15 min prior to measurements. *Via* the IMAGING PAM MAXI (Walz, Effeltrich, Germany), the images of fluorescence emission were digitized within the camera and transmitted *via* a Firewire interface (400 megabits/s; Firewire-1394, Austin, TX, United States) to a personal computer for storage and analysis. Measurements and calculations of ETR, ΔF/F_m_′, and NPQ were determined according to He et al. (2011).

### Determination of CAM Acidity

Based on He and Teo (2007), leaf discs were punched from fully expanded young leaves and then placed in microtitre plate wells before the beginning and the end of photoperiod. The Milli-Q water (1 ml) was added to each well before placing in a 95°C water bath for 15 min. The extracts in the wells were titrated against 0.005 M NaOH, using three drops of phenolphthalein for indicator until end-point was reached. Final volume of NaOH used to reach end-point was used to calculate CAM acidity as both μmol H^+^ g^−1^ FW and μmol H^+^ g^−1^ DW.

### Measurements of Light Response Curves of Net Photosynthetic O_2_ Evolution Rate (P_N_), PS II, and Cyt b_6_f Concentrations

According to He and Qin (2020b), modified from He and Chow (2003), these parameters were measured at 25°C from leaf discs of fully expanded young leaves, which were harvested 16–24 days after transplanting. A whole leaf as placed in a gas-phase oxygen electrode (Hansatech, King’s Lynn, UK) chamber contained 1% CO_2_ supplied by 1 M NaHCO_3_/Na_2_CO_3_ (pH 9). Two illumination regimes were used to obtain photosynthetic O_2_ evolution rates: (1) repetitive flash illumination with saturating, single-turnover flashes or (2) continuous white light from light emitting diodes. The measurements of P_N_ and the calculations of PS II and Cyt *b_6_f* concentrations were carried out as in Zhu et al. (2017) and He and Qin (2020b).

### Measurement of NO_3_^−^ Concentration

Dried tissues of 0.01 g were grounded with Mill-Q water, and the details of extraction processes were described in He et al. (2020). The flow injection analyzer (Model Quickchem 800, Lachat Instruments Inc., Milwaukee, United States) was used to determine NO_3_^–^ concentration according to He et al. (2020).

### Measurement of Total Reduced Nitrogen

Dried shoot tissues (0.05 g) were digested with a Kjeldahl tablet in concentrated sulfuric acid for 60 min at 350°C (Allen, 1989), and the mixture was allowed to cool before TRN was determined by a Kjeltec 2300 analyzer (Foss Tecator AB, Höganäs, Sweden) through titration.

### Measurements of Leaf Total Soluble Protein and Rubisco Protein Contents

Frozen leaf samples of 1 g were ground in liquid nitrogen. The details of extraction processes were described in He et al. (2017). The amount of TSP was determined using the method according to Lowry et al. (1951). Rubisco protein present in the samples was quantified using SDS-PAGE. Solubilized protein was boiled for 5 min and loaded onto the Mini-PROTEAN Precast Gel (TGX gel, any kD, BIO-RAD, United States) to run the gels for 30 min and then stained for 1 h using coomassie brilliant blue according to He et al. (2017). The separated proteins stained were analyzed using a Fluor Chem 8800 gel imagine system under visible light. The amount of TSP loaded were used to calculate Rubisco content based on the band of large subunit (LSU) and small subunit (SSU), and Rubisco content is the sum of LSU and SSU.

### Determination of Total Soluble Sugars

After 30 days of transplanting, leaf tissue was dried at 80°C for 4 days. Dry samples of 10 mg were used to extract TSS using hot 80% ethanol. The details of extraction processes were described in He et al. (2020). The concentration of free soluble sugar was determined colorimetrically at 490 nm using a spectrophotometer (UV-2550 Shimadzu, Japan) according to Dubois et al. (1956).

### Determination of Proline Concentration

This assay was modified from the protocol by Bates et al. (1973) using frozen leaf tissues of 0.5 g. The details of extraction processes and the measurements of absorbance at 520 nm using a spectrophotometer (UV-2550 Shimadzu, Japan) were described in He et al. (2020).

### Measurement of Ascorbic Acids

Total ascorbic acid (Asc) was assayed from 0.5 g of frozen leaves harvested from fully expanded young leaves 33 days after transplanting by the reduction of 2,6-dichlorophenolindophenol (DCPIP) according to Leipner et al. (1997) and modified by He et al. (2020). The Asc concentrations were spectrophotometrically assayed by measuring the absorbance at 524 nm using a spectrophotometer (UV-2550 Shimadzu, Japan). L-ascorbic acid was used as a standard.

### Determination of Total Phenolic Compounds

Frozen leaf samples (0.5 g) were ground in liquid nitrogen and 5 ml of 80% methanol (Ragaee et al., 2006). The details of extraction processes and the measurements of absorbances at 765 nm using a spectrophotometer (UV-2550 Shimadzu, Japan) were described in He et al. (2020).

### Determination of Dietary Minerals

Dried tissues were digested in 65% nitric acid using an UltraWAVE single reaction chamber microwave digestion system (Milestone, United States). Digested samples were diluted with Milli-Q water. The dietary minerals were measured using Optima 8,300 ICP-OES Spectrometer and WinLab 32 (Perkin Elmer, United States) according to Bocchini et al. (2015).

### Statistical Analysis

One-way ANOVA was used to test for significant differences of different variances crossed with the four salinity treatments. LSD multiple comparison tests were used to discriminate the means (IBM SPSS Statistics 25).

## Results

### Shoot and Root Productivity

**Figure 2:**
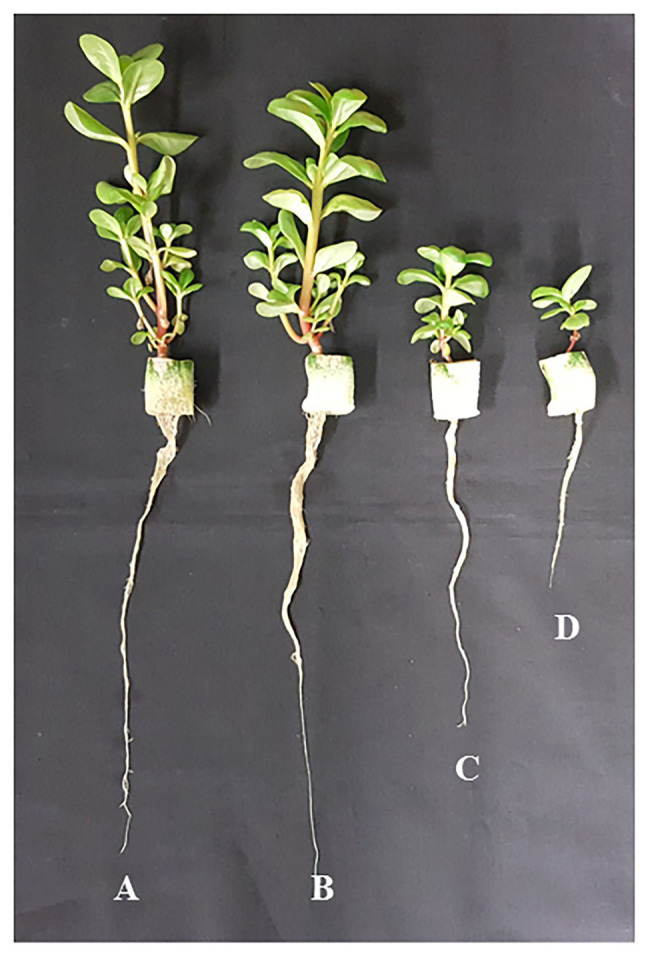
Purslane plants grown indoors hydroponically with 0 **(A)**, 100 **(B)**, 200 **(C)**, and 300 mM NaCl **(D)** under LED lighting (R/B ratio of 2.2) for 30 days.

**Figure 3:**
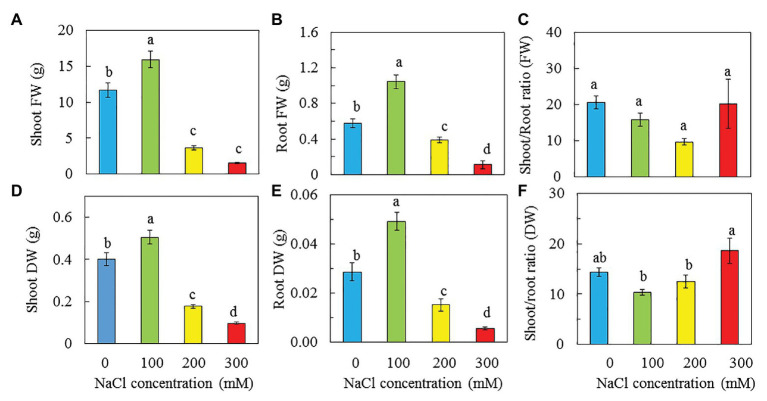
Shoot FW and DW **(A,D)**, root FW and DW **(B,E)**, and shoot/root ratio FW and DW **(C,F)** of *P. oleracea* grown hydroponically with different NaCl salinities for 30 days. Vertical bars represent the standard errors. Means with different letters are statistically different (*p* < 0.05; *n* = 5) as determined by LSD multiple comparison test.

All purslane plants grew well and appeared healthy from transplanting to harvest although plants grown with 200 and 300 mM NaCl were much smaller compared to those grown with 0 and 100 mM NaCl (Figure 2). At harvest, the shoot, root FW, and DW of purslane plants were the highest with 100 mM NaCl than with 0, 200, and 300 mM NaCl. Purslane plants with 100 mM had shoot FW almost 10-fold higher than those grown with 300 mM. Statistically, there was no difference in shoot FW between purslane grown with 200 and 300 mM NaCl but they were significantly lower than those grown with 0 mM NaCl (Figure 3A). For shoot DW (Figure 3D), root FW (Figure 3B) and root DW (Figure 3E), purslane grown with 100 mM NaCl had the highest values followed by those grown with 0 mM NaCl and then 200 mM NaCl while those grown with 300 mM NaCl had the lowest value. There were no significant differences in shoot/root ratio FW among different treatments (Figure 3C). For shoot/root ratio DW, plants grown with 300 mM NaCl had the highest value followed by those with 0 mM NaCl and those with 100 and 200 mM NaCl had similar lowest readings (Figure 3F).

### Leaf Growth and Leaf Water Status

**Figure 4:**
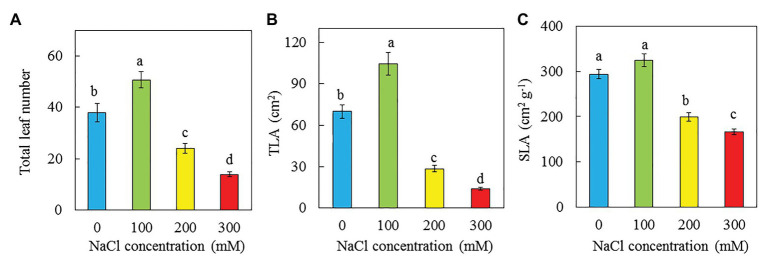
Total leaf number **(A)**, TLA **(B)**, and SLA **(C)** of *P. oleracea* grown hydroponically with different NaCl salinities for 30 days. Vertical bars represent the standard errors. Means with different letters are statistically different (*p* < 0.05; *n* = 5) as determined by LSD multiple comparison test.

**Figure 5:**
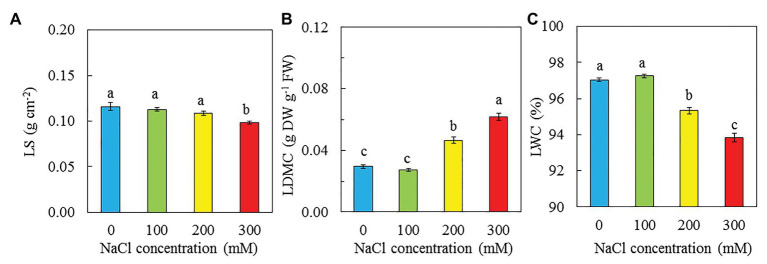
LS **(A)**, LDMC **(B)**, and LWC **(C)** of *P. oleracea* grown hydroponically with different NaCl salinities for 30 days. Vertical bars represent the standard errors. Means with different letters are statistically different (*p* < 0.05; *n* = 5) as determined by LSD multiple comparison test.

Purslane grown with 100 mM NaCl had the highest total leaf number and TLA followed by those grown with 0 and then 200 mM NaCl while those grown with 300 mM NaCl had the lowest value (Figures 4A,B). There were no significant difference in SLA between plants grown with 0 and 100 mM NaCl but they were significantly higher than those grown with 200 and 300 mM NaCl. Plants grown with 300 had the lowest SLA (Figure 4C). There were no significant differences in LS among plants grown with 0, 100, and 200 mM NaCl, and they were significantly higher than that of plants grown with 300 mM NaCl (Figure 5A). The trend for LWC was similar to that of LS except for those grown with 200 mM NaCl had significantly lower LWC than with 0 and 100 mM NaCl (Figure 5C). LDMC increased with increasing NaCl concentrations. Plants grown with 300 had the highest LDMC while those with 0 and 100 mM had the lowest values (Figure 5B).

### Photosynthetic Pigments

**Figure 6:**
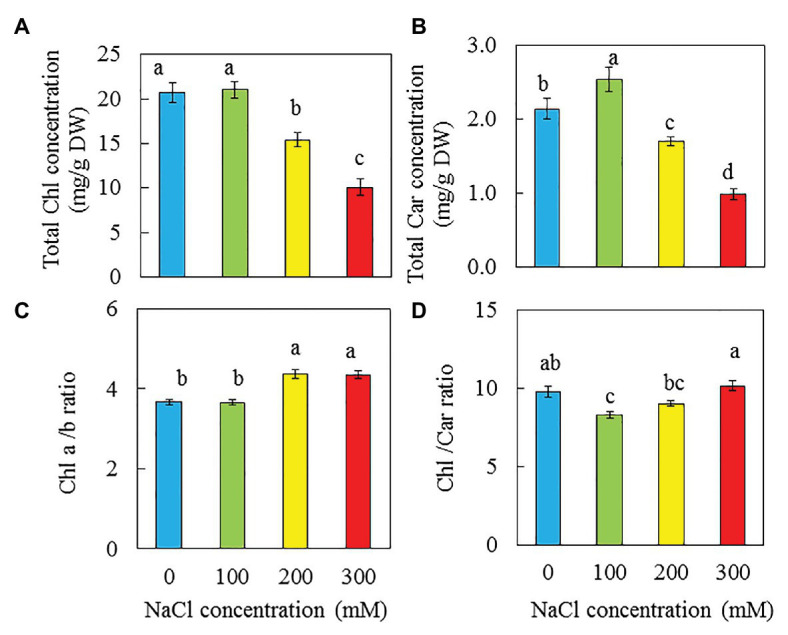
Total Chl concentration **(A)**, total Car concentration **(B)**, Chl a/b ratio **(C)**, and Chl/Car ratio **(D)** of *P. oleracea* grown hydroponically with different NaCl salinities for 26 days. Vertical bars represent the standard errors. Means with different letters are statistically different (*p* < 0.05; *n* = 4) as determined by LSD multiple comparison test.

There were no significant difference in total Chl concentration between purslane grown with 0 and 100 mM NaCl but they were significantly higher than those grown with 200 and 300 mM NaCl. Purslane plants grown with 200 mM had higher total Chl concentration than with 300 mM NaCl (Figure 6A). For total Car concentration, purslane grown with 100 mM NaCl had the highest value followed by those grown with 0 mM NaCl and then 200 mM NaCl while those grown with 300 mM NaCl had the lowest reading (Figure 6B). Chl a/b ratios were significantly higher in plants grown with 200 and 300 mM NaCl compared to those grown with 0 and 100 mM NaCl (Figure 6C). Purslane grown with 100 mM had the lowest Chl/Car ratio due to its highest Car concentration among the different treatments (Figure 6D).

### F_v_/F_m_ Ratio, ETR, ΔF/F_m_', and NPQ

**Figure 7:**
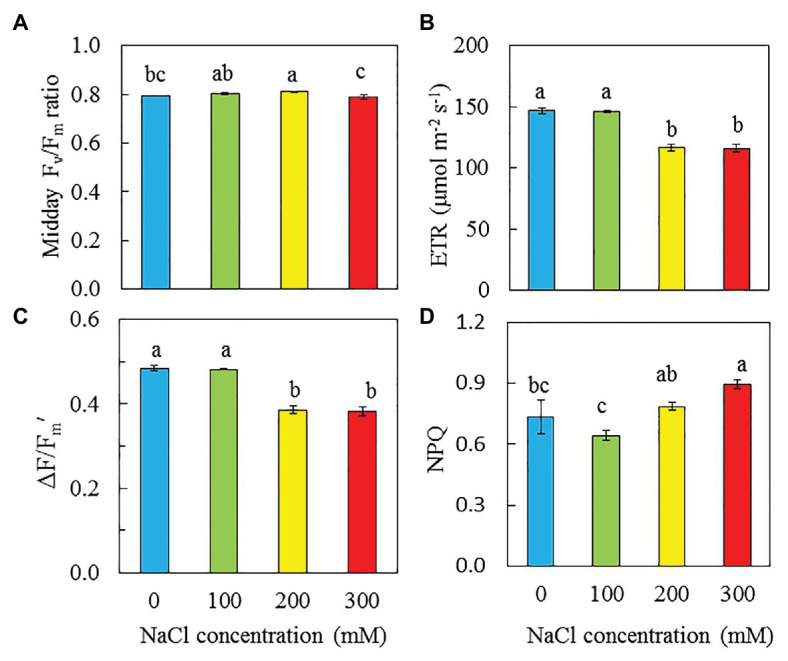
Midday Chl fluorescence F_v_/F_m_ ratio **(A)**, ETR **(B)**, ∆F/F_m_′ **(C)**, and NPQ **(D)** of *P. oleracea* grown hydroponically with different NaCl salinities for 26 days. Vertical bars represent the standard errors. Means with different letters are statistically different (*p* < 0.05; *n* = 5 for A; *n* = 6 for B-D) as determined by LSD multiple comparison test.

Figure 7 shows the maximum quantum yield of PSII, measured by F_v_/F_m_ ratio and photochemical light use efficiency measured by ETR, ΔF/F_m_′, and NPQ under an actinic light of 606 μmol m^−2^ s^−1^, which was the saturating irradiance for ETR. Although there were difference in F_v_/F_m_ ratios statistically, all readings were close or slightly higher than 0.8 (Figure 7A). The values of ETR and ΔF/F_m_' were significantly higher in purslane grown with 0 and 100 mM NaCl compared to those grown with 200 and 300 mM NaCl (Figures 7B,C). Purslane grown with 300 mM NaCl had the highest NPQ followed by those grown with 200 mM and then 0 and 100 mM conditions (Figure 7D).

### Light Response Curves of P_N_ and CAM Acidity

**Figure 8:**
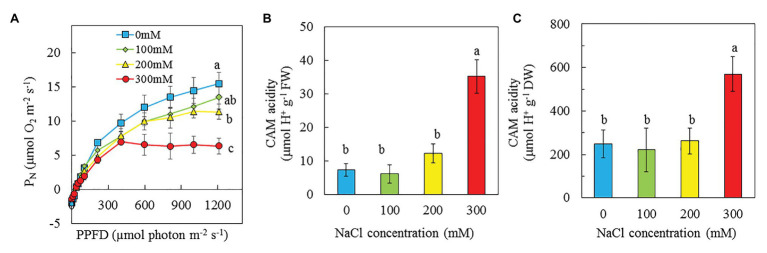
Light response curves of P_N_ **(A)**, CAM acidity on a FW basis **(B)**, and CAM acidity on a DW basis **(C)** of *P. oleracea* grown hydroponically with different NaCl salinities from 16 to 24 days (A) and for 30 days (B,C) after transplanting. Vertical bars represent the standard errors. Means with different letters are statistically different (*p* < 0.05; *n* = 5) as determined by LSD multiple comparison test.

Light response curves of P_N_ were determined from all plants grown under different NaCl salinities. The values of P_N_ for purslane plants grown with 0 and 100 mM NaCl increased with increasing PPFD from 10 to 1,210 μmol m^−2^ s^−1^. However, P_N_ of purslane grown with 300 and 200 mM NaCl reached saturated points, respectively, at PPFD of 405 and 808 μmol m^−2^ s^−1^ (Figure 8A). Measured under the highest PPFD, P_N_ of purslane plants grown with 0 mM NaCl was not significantly different from those grown with 100 mM but significantly higher than that of plants grown with 200 and 300 mM NaCl. Although there was no significant difference in P_N_ between 100 and 200 mM, they were both significantly higher than that of plants grow with 300 mM NaCl (Figure 8A). There were no significant differences for CAM acidity among plants grown with 0, 100, and 200 mM NaCl. However, the CAM acidities on the basis of FW and DW for purslane grown with 300 mM were respectively about 4.5- and 2.5-fold higher than the rest plants (Figures 8B,C).

### PS II and Cyt *b_6_f* Concentrations

**Figure 9:**
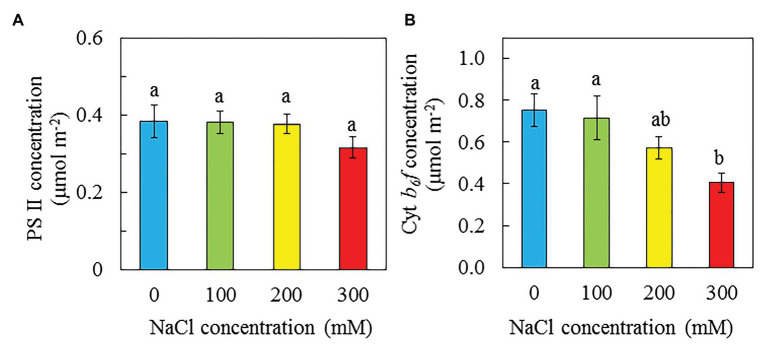
PS II concentration **(A)** and Cyt *b_6_f* concentration **(B)** of *P. oleracea* grown hydroponically with different NaCl salinities from 16 to 24 days after transplanting. Vertical bars represent the standard errors. Means with different letters are statistically different (*p* < 0.05; *n* = 4) as determined by LSD multiple comparison test.

PS II concentrations seem to be lower in plants grown with 300 mM NaCl compared to other conditions. However, statistically, there were no significant differences in PS II concentrations among purslane grown with different NaCl salinities (Figure 9A). For Cyt *b_6_f* concentrations, they were similar but significantly higher in plants grown with 0 and 100 compared to those with 300 mM NaCl. There was no significant different in Cyt *b_6_f* between purslane grown with 200 and 300 mM NaCl. Cyt *b_6_f* concentrations for plants grown with 0, 100, and 200 mM NaCl were respectively 46, 43, and 29% greater than those of plants grown with 300 mM NaCl (Figure 9B).

### NO_3_^−^, TRN, Total Leaf Soluble Protein, and Rubisco Protein

**Figure 10:**
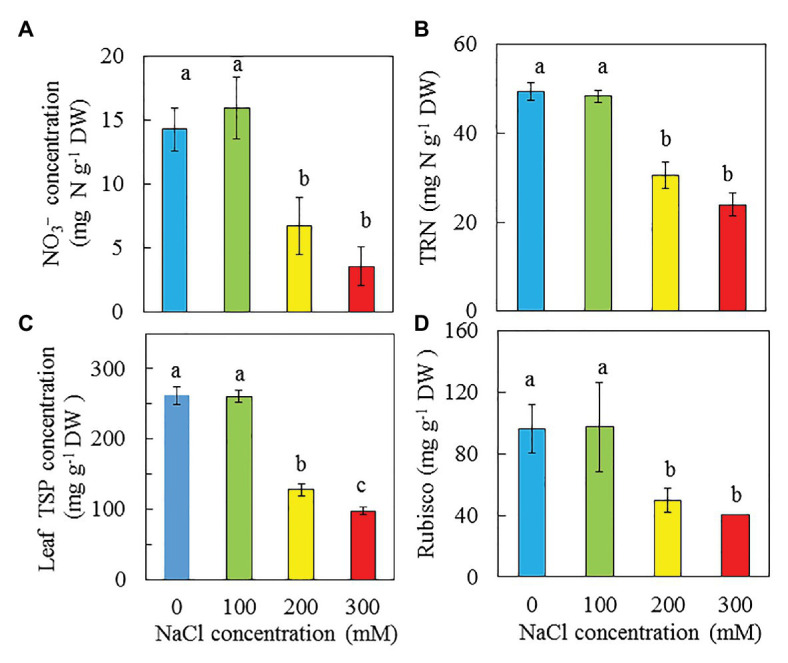
Concentrations of NO_3_^−^ **(A)**, TRN **(B)**, total soluble protein **(C)**, and Rubisco **(D)** in the leaves of *P. oleracea* grown hydroponically with different NaCl salinities for 30 days (A,B) and 33 days (C,D), respectively. Vertical bars represent the standard errors. Means with different letters are statistically different (*p* < 0.05; *n* = 3) as determined by LSD multiple comparison test.

On a DW basis, the concentrations of NO_3_^−^, TRN, and Rubisco protein exhibit similar trends in response to NaCl salinity, showing that purslane plants grown with 0 and 100 mM NaCl were similar but significantly higher than those grown with 200 and 300 mM NaCl (Figures 10A,B,D). For leaf total soluble protein, the plants grown with 0 and 100 mM NaCl had the highest values followed by those grown with 200 mM NaCl while plants grown with 300 mM Nacl had the lowest concentration (Figure 10C).

### Proline, TSS, Asc, and Total Phenolic Compounds

**Figure 11:**
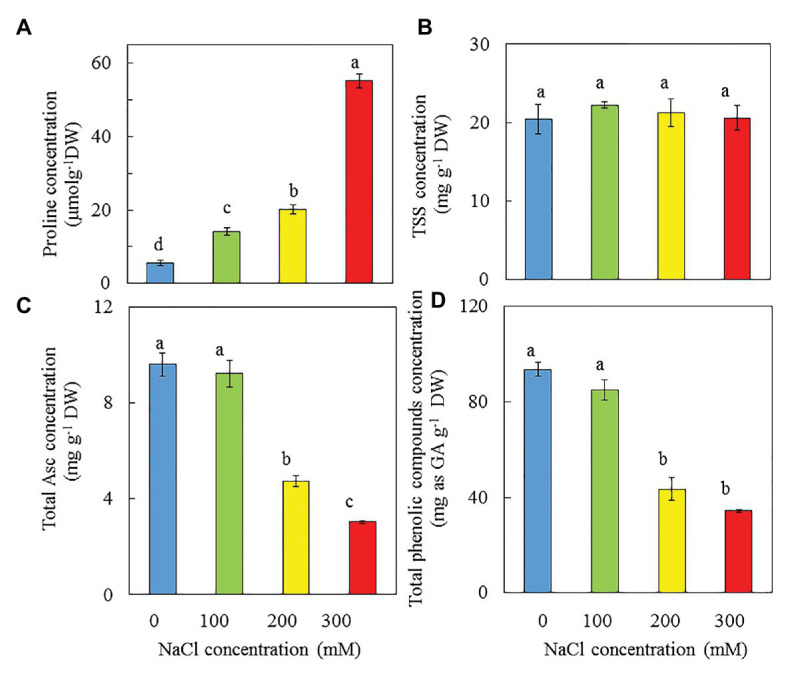
Concentrations of proline **(A)**, TSS **(B)**, total Asc **(C)**, and total phenolic compounds **(D)** in the leaves of *P. oleracea* grown hydroponically with different NaCl salinities for 30 days (B) and 33 days (A,C,D), respectively. Vertical bars represent the standard errors. Means with different letters are statistically different (*p* < 0.05; *n* = 3) as determined by LSD multiple comparison test.

Purslane grown with 0 mM NaCl had the lowest proline concentration, which increased with increasing NaCl salinity. Plants grown with 300 mM NaCl had the highest proline concentration, which was 2.7-, 3.9-, and 10-fold of those respectively grown with 200, 100, and 0 mM NaCl (Figure 11A). However, there were no significant differences in TSS concentration among all plants (Figure 11B). Purslane plants grown with 0 and 100 mM NaCl produced much higher total Asc (Figure 11C) and total phenolic compounds (Figure 11D) compared to those grown with 200 and 300 mM NaCl. Similar highest Asc concentrations were obtained from plants grown with 0 and 100 mM NaCl and they were 98 and 210%, respectively, higher than those grown with 200 and 300 mM NaCl. Purslane grown with 0 and 100 mM NaCl also had similar highest concentration of total phenolic compounds and they were 105 and 160%, respectively, higher than those of plants grown with 200 and 300 mM NaCl (Figure 11D).

### Dietary Minerals

**Figure 12:**
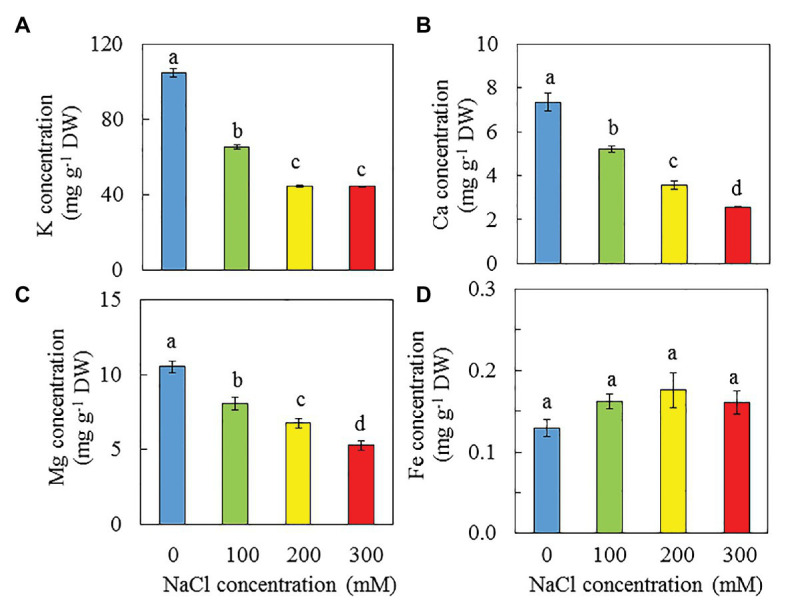
K **(A)**, Ca **(B)**, Mg **(C)**, and Fe **(D)** concentrations *n* in the leaves of *P. oleracea* grown hydroponically with different salinities for 30 days. Vertical bars represent the standard errors. Means with different letters are statistically different (*p* < 0.05; *n* = 5) as determined by LSD multiple comparison test.

High NaCl salinity resulted in decreases of K, Ca, and Mg (Figures 12A–C). The concentrations of K, Ca, and Mg were highest in purslane grown with 0 mM NaCl followed by those grown with 100 mM and then 200 mM NaCl while those grown with 300 mM NaCl had the lowest concentration. Statistically, there were no significant differences in Fe concentrations among all treatments. However, salinity seems to increase Fe concentration of purslane (Figure 12D).

## Discussion

### Productivity of Shoot and Root, Leaf Growth, and Leaf Water Status

As common purslane is a drought and salinity tolerant plant (Yazici et al., 2007; Teixeira and Carvalho, 2009), it is an important crop for farming in the Mediterranean basin. According to Alam et al. (2014a), it can also be grown under various soil conditions and types at different locations of West Peninsular, Malaysia. In this study, purslane plants were grown hydroponically indoors with different NaCl salinities under a constant light intensity of 200 μmol m^−2^ s^−1^ provided by LEDs and all plants appeared healthy (Figure 2). Plants grown with 100 mM NaCl had the highest shoot and root biomass compared to those with 0, 200, and 300 mM NaCl (Figure 3). According to Flowers et al. (1986), most halophytes require saline conditions to attain optimal growth. Our results showed that purslane plants grown with 100 mM NaCl had higher shoot and root productivity compared to those grown with fresh water (0 mM NaCl). Furthermore, according to our observation, purslane plants have the ability to complete their lifecycle on >200 mM NaCl, and thus it is a halophyte crop (Yuan et al., 2019). Grown with different NaCl salinities indoors hydroponically under LED lighting, our study also showed that an editable halophyte *M. crystallinum* L. required 100 mM NaCl to perform better growth compared to those grown with fresh water (He and Qin, 2020a). However, high salt concentration often results in hyperosmotic and oxidative stress, which can hinder the growth and development of plants, and may even lead to death (Xiong et al., 2002; Fan et al., 2011). Although those grown with higher salinities, such as 200 and 300 mM NaCl, were much smaller compared to those grown with 0 and 100 mM NaCl (Figures 2, 3), none of them showed any visual symptoms of damage, indicating that the purslane plants in this study had a capacity to withstand soil salinity up to 300 mM. Similarly, cultivating purslane under 240 mM NaCl hydroponically, Anastaćio and Carvalho (2013) did not observe any toxicity symptoms. It was reported that the salinity tolerance of purslane and the impact of salinity on purslane growth and development depend on other conditions such as genotypes (Alam et al., 2014b) and light level (Franco et al., 2011). Moreover, Poljakoff-Mayber and Lerner (1999) reported that shoot growth was frequently inhibited more than root growth under saline conditions. However, with purslane, Franco et al. (2011) found the opposite, with greater reductions in root length than in stem length. In the study with *M. crystallinum* grown with different NaCl salinities aeroponically indoors under LED lighting, we have recently found that shoot/root ratio FW and DW were, respectively, the lowest and the highest in *M. crystallinum* grown with 500 mM NaCl compared to those grown with 100 and 250 mM NaCl. The different responses to salinity between shoot/root ratio FW and DW resulted from the lowest LWC and highest LDMC in *M. crystallinum* grown under the highest salinity of 500 mM NaCl (unpublished). In this study with purslane, all plants had similar shoot/root ratio FW (Figure 3C). However, plants grown with 300 mM NaCl had the highest shoot/root DW (Figure 3F) due to its highest LDMC (Figure 5B) and lowest LWC (Figure 5C).

Low productivity of purslane under higher salinity, such as 200 and 300 mM NaCl, could mainly be due to its slow leaf growth and development supported by the lower total number of leaves (Figure 4A) and smaller TLA (Figure 4B). In the study with two purslane genotypes, Zaman et al. (2019) reported that leaf numbers were significantly decreased in the main stem in both genotypes when grown with high concentration of NaCl due to excess accumulation of salt during the development of leaf. This study also showed that plants grown with 0 and 100 mM NaCl had similar but significantly higher SLA compared to those grown with 200 and 300 mM NaCl (Figure 4C). The SLA was the lowest in purslane grown with 300 mM NaCl (Figure 4C), resulting from its lowest TLA (Figure 4B) and highest LDMC (Figure 5B). According to Rodríguez et al. (2005), decreases in leaf area linked to salinity directly affect the SLA of *Asteriscus maritimus* plants as salinity increased. In study with *M. crystallinumm*, we found that plants grown with higher salinity had lower TLA and lower SLA (He and Qin, 2020a). Similar results were observed in this study with purslane plants (Figures 4A,B). The decrease of SLA is often associated with the increase in LS of salt tolerant species (Geissler et al., 2009; de Vos et al., 2010, 2013; Rozema et al., 2015). LS is measured as the maximum water content expressed as fresh mass per unit of leaf area (g FW cm^−2^; Debez et al., 2004). In our study of *M. crystallinumm*, plants with 250 and 500 mM NaCl had much lower SLA compared to those grown with 0 and 100 mM NaCl while salinity did not affect its LS on a leaf area basis (He and Qin, 2020a). In this study, purslane grown with 300 mM NaCl had lower SLA and LS compared to those grown with other lower concentration of NaCl (Figures 4C, 5A). The lower LS of purslane grown with higher salinity (Figure 5A) could be partially due to its lower LWC (Figure 5C). According to Belkheiri and Mulas (2013), LS increased with increasing of salinity. Optimal NaCl concentration for growth has been reported to be the concentration at which LS is highest. A further increase in salinity resulted in decrease of both growth and LS (Khan et al., 2000). However, it appears that the correction between SLA and LS depends on plant species.

### Photosynthetic Pigments

Salinity stress could affect the photosynthetic performance due to salt accumulation in leaves (Munns and Tester, 2008) and the deceases in total Chl (Parida et al., 2002; Stepien and Johnson, 2009; Duarte et al., 2013; Zaman et al., 2019). In the study of the glycophyte Arabidopsis and the halophyte Thellungiella, Stepien and Johnson (2009) found that the Chl content of Arabidopsis continuously declined when exposed to salt while there was no significant change in the Chl content of Thellungiella when exposed to same salinity conditions. In our recent study with *M. crystallinumm*, it was found that plants grown with 500 mM NaCl had significantly higher total Chl content than those grown with 0, 100, and 250 mM NaCl (unpublished). Similarly, in the study with two obligate halophytes, *Sesuvium portulacastrum* and *Tecticornia indica*, total Chl contents were significantly enhanced in both species grown under 200 mM and 400 mM NaCl (Rabhi et al., 2012). In the study with two purslane genotypes, Zaman et al. (2019) found that with the increase of NaCl concentrations from 0 to 200 mM, total Chl content decreased. In this study, higher salinity also resulted in lower Chl content in purslane (Figure 6A). Apparently, salt-tolerant species show increased or unchanged Chl content under higher salinity conditions, whereas Chl levels decrease in salt-sensitive species. In the study with purslane, Franco et al. (2011) reported that salinity did not affect the Chl content when it was grown in the growth chambers under lower level of light (73.5 μmol m^−2^ s^−1^). However, in the same study with purslane, it was found that the Chl content decreased with an increase in salinity when they were grown in the greenhouse under higher light intensity (530 μmol m^−2^ s^−1^). Based on the above discussion, the total Chl content could be considered as a biochemical marker of salt tolerance in plants (Stepien and Johnson, 2009) although the impact of salinity on total Chl content may depend on light intensity (Franco et al., 2011). In the study with halophyte, *Plantago coronopus* (L.), Koyro (2006) reported that salt-induced increase of the Car content, which could function to dissipate the excess energy in the PS I and PS II. In this study, opposite result was obtained for purslane as significantly decreased total Car concentration was observed in purslane grown with 300 mM NaCl while those grown with 100 mM NaCl had the highest value (Figure 6B). The Chl a/b ratios of purslane was also affected by salinity, showing a significant increase when grown with 200 and 300 mM NaCl (Figure 6C). However, the lowest Chl/Car (or the highest Car/Chl) ratio was observed in plant grown with 100 mM NaCl (Figure 6D). Chl b is mainly located in the light harvesting complex, the higher Chl a/b ratio in purslane grown with higher salinity could result in reduced photosynthetic light use efficiency (Koyro, 2006).

### Photosynthetic Performance

Chl fluorescence could provide information about the performance of PS II (Maxwell and Johnson, 2000). Maximal efficiency of PS II photochemistry measured by Chl fluorescence F_v_/F_m_ ratio from the dark-adapted leaves is an early indicator of salinity stress (Broetto et al., 2007; Matsuoka et al., 2018; Zaman et al., 2019). In the study with two purslane genotypes, Zaman et al. (2019) reported that with the increase of NaCl concentrations, F_v_/F_m_ ratio significantly decreased in both genotypes. However, in this study, F_v_/F_m_ ratios in all dark-adapted purslane leaves were close to 0.8 (Figure 7A), suggesting that there was no evidence of damage to PS II in any plants (Broetto et al., 2007). The different responses of F_v_/F_m_ ratios of purslane plants to salinity between this study and the study by Zaman et al. (2019) could be due to the different growth conditions. In this study, all plants were grown indoors under a PPFD of 200 μmol m^−2^ s^−1^ while Zaman et al. (2019) cultivated their plants in the greenhouse under a PPFD of 400 μmol m^−2^ s^−1^. Our recent study with *M. crystallinum* grown indoors under a 200 μmol m^−2^ s^−1^ also showed that salinity stress did not affect the maximal efficiency of PS II photochemistry (He and Qin, 2020a). While the impacts of salinity on maximal efficiency of PS II photochemistry may depend on the light level under which halophytes were grown, general decreases in ETR, qP, and the effective quantum yield of PS II (ΔF/F_m_'), but increases in NPQ have been reported under salt stress conditions (Broetto et al., 2007; D’Andrea et al., 2014; Acosta-Motos et al., 2017). Decrease in PS II efficiency and increase in NPQ correlated with the response of plants to salt stress could be a strategy to safely dissipate excess energy (Acosta-Motos et al., 2017). Study with purslane plants, D’Andrea et al. (2014) found that drought stress resulted in decreases of ETR and ΔF/F_m_′ and an increase of NPQ. In this study, decreases in ETR and ΔF/F_m_' were observed in purslane grown with 200 and 300 mM NaCl compared to those with 0 and 100 mM NaCl (Figures 7B,C). It was reported that PS II and Cyt *b_6_f* may be the sites of the rate-limiting step in the electron transport chain (Eichelmann et al., 2000). Stepien and Johnson (2009) reported that the inhibition of linear electron flow in Arabidopsis under salt stress was accompanied by a downregulation of electron flow through the Cyt *b_6_f* complex. In this study, lower ETR and ΔF/F_m_′ observed in purslane grown with high salinity could be due to the tuning of the amount of active PSII reaction centers and regulating the electron transfer by the Cyt *b_6_f* complex (Tikkanen et al., 2012; He and Qin, 2020b). This was supported by the results of lower PS II (Figure 9A) and Cyt *b_6_f* (Figure 9B) concentrations in purslane grown with 300 mM NaCl compared to other conditions. It was also found that purslane grown with the highest NaCl salinity had the highest NPQ (Figure 7D). Increased NPQ implies that purslane grown with the highest salinity such as 300 mM NaCl in this study could safely dissipate excess energy (Acosta-Motos et al., 2017). This also explains why purslane plant grown with high concentration of NaCl was healthy regardless of its slow growth (Figure 2).

Acosta-Motos et al. (2015) concluded that the drop in P_N_ paralleled with a decline in the effective quantum yield of PS II (ΔF/F_m_′) and increases in NPQ, acted as a safe mechanism for dissipating excess light energy. In this study, P_N_ of purslane grown with higher salinity reached saturated points at lower PPFDs. Measured under the highest PPFD of 1,210 μmol m^−2^ s^−1^, P_N_ of purslane plants grown with 300 mM was much lower compared to those grown with lower NaCl concentrations (Figure 8A). The reduction of P_N_ could also be due to the decrease of Calvin Cycle enzymes such as Rubisco (Acosta-Motos et al., 2017). It has been reported that salt stress alter Rubisco expression (Dionisio-Sese and Tobita, 2000). In the study with *Desmostachya bipinnata* (L.) Staph, Asrar et al. (2017) found that decreased Rubisco content was observed with increasing salinity treatments. In this study, purslane plants grown with 200 and 300 mM NaCl had much lower soluble protein and Rubisco protein compared to those grown with 0 and 100 mM NaCl (Figures 10C,D).

CAM is normally elicited in *purslane* under drought stress (Lara et al., 2004; D’Andrea et al., 2014; Winter and Holtum, 2014; Mulry et al., 2015). Salinity and drought are two often simultaneously occurring stresses. Thus, CAM acidity was also included in this study. In the study with *M. crystallinum*, Borland et al. (2006) concluded that CAM can be induced by abiotic and biotic stresses in responses to changing environmental conditions. Cushman et al. (2008) suggested that CAM acidity levels of *M. crystallinumm*, at least 40 μmol H^+^ g^−1^ FW were deemed to be performing CAM under saline conditions. In this study, on the basis of both FW and DW, the CAM acidities for purslane grown with 300 mM were much higher compared to the rest of plants (Figures 8B,C). Although CAM acidity of purslane grown with 300 mM was slightly lower than 40 μmol H^+^ g^−1^ FW (Figure 8B), it was most likely be engaging in CAM-like under high salt stress as it was a 4.5-fold increase compared to those grown with lower concentrations of NaCl. The term “CAM-like” has been used for purslane plants (Lara et al., 2003). *Portulaca* spp. tend to inhabit environments with high light intensities, weak CAM acidity of purslane plants under high salinity in this study could be due to the low level of light used to cultivate this plant species. Furthermore, there are different CAM expression levels within the *P. oleracea* complex (Ferrari et al., 2020). Under drought stress, when CAM was induced in purslane, lower qP, and higher NPQ were observed (D’Andrea et al., 2014). In this study, CAM-like purslane grown under the highest salinity of 300 mM NaCl also had higher NPQ (Figure 7D). The regulation of NPQ determines the levels of plant responses under saline conditions. Studies have showed that higher NPQ was observed in CAM-inducible *M. crystalline* under high salt stress (Broetto et al., 2007; Niewiadomska et al., 2011; Matsuoka et al., 2018). Thus, increased NPQ under high salinity condition can also be one of the indicators for CAM induction.

### Nutritional Quality

Proline is a metabolite with multiple roles such as antioxidant and osmoprotectant (Szabados and Savouré, 2010). Thus, proline that can be utilized in functional food is produced for protection against hyperosmotic stress caused by high salinity (Flowers and Colmer, 2008; Agarie et al., 2009; Benjamin et al., 2019; Hsouna et al., 2020). It has been reported that the correlation between saline concentration and the level of proline gene expression in purslane varies by population (Sdouga et al., 2019). In this study, the accumulation of proline in purslane plants increased with increasing salinity (Figure 11A). Yazici et al. (2007) also reported that salt stress resulted in high accumulation of proline in purslane. TSS is also well-known osmolyte that enables plants to avoid the consequences of hyperosmotic stress (Hsouna et al., 2020). In our recent study, higher TSS accumulation was observed in *M. crystallinum* grown with 500 mM NaCl compared to those grown with lower concentration of NaCl (unpublished). However, in this study, all purslane plants had similar level of TSS (Figure 11B). The natural antioxidants, such as Asc and phenolic compounds, play important roles in various physiological responses to stresses in purslane plants (Lim and Quah, 2007; Uddin et al., 2012a). There are very few studies available on the impact of salinity on the contents of Asc and phenolic compounds in purslane grown under different salinity conditions indoors with hydroponic cultivation. High levels of vitamin C and some vitamins of complex-B have also been identified in purslane (Petropoulos et al., 2016). In this study, Asc concentration of purslane grown with 0 and 100 mM NaCl were 98 and 210%, respectively, higher than those grown with 200 and 300 mM NaCl (Figure 11C). These results indicate that increasing NaCl concentration from 100 mM to 200 or 300 mM did not increase the Asc concentration. Our recent studies have shown that drought stress enhanced the concentrations of Asc of *M. crystallinum* grown indoors under combination of red and blue-LED lighting (He et al., 2020). In another study, we also found that Asc concentration of *M. crystallinum* increased with increasing NaCl from 0 to 500 mM NaCl (unpublished data). It was reported that the antioxidant potential of purslane mainly depends on the content of total phenolic compounds (Uddin et al., 2012a). The contents of total phenolic compounds in different plant tissues increased with the increase in salinity have also been reported in a number of plants (Agastian et al., 2000; Muthukumarasamy et al., 2000; Navarro et al., 2006). In the study with halophyte *Cakile maritima*, Ksouri et al. (2007) concluded that plants, such as halophytes tolerant to stress, are potentially interesting systems for production of secondary metabolites, such as phenolic compounds, are useful for food and medicinal applications. In this study, it was unexpected to see that purslane plants grown with 0 and 100 mM NaCl accumulated much higher total phenolic compounds compared to those grown with 200 and 300 mM NaCl (Figure 11D). This could be due to the fact that plants vary widely in their concentrations of total phenolic compounds with both genetics and environment affecting the level of phenolic compounds (De Abreu and Mazzafera, 2005). Lim and Quah (2007) reported that there is a great variation in the accumulation of total phenolic compounds, which was directly dependent on the season of the year under natural conditions. Furthermore, lower concentration of Asc and total phenolic compounds for purslane grown under higher salinity could be associated with their lower photosynthetic performance (Figures 7B,C, 8A). In the study with *Hypericum perforatum* L. (St. John’s wort), a traditional herb, Mosaleeyanon et al. (2005) reported that growing this herb plants under optimal conditions can enhance biomass and secondary metabolite production by increasing net photosynthetic rate. Establishing optimal growth conditions to enhance photosynthetic capacity and secondary metabolites of purslane grown indoors with different saline conditions merit our future study.

According to Corrè and Breimer (1979), purslane has very high NO_3_^−^ content (>2,500 mg kg ^−1^ FW). In this study, the NO_3_^−^ concentrations on a DW basis of purslane plants grown with 0 and 100 mM NaCl were similarly and significantly higher (about 2 folds) than those grown with 200 and 300 mM NaCl (Figure 10A). Lower NO_3_^−^ concentrations are associated with lower concentrations of TNR, total soluble protein and Rubisco protein (Figures 10B–D). Lower total Chl content in the leaves of purslane grown with higher concentrations of NaCl could be partially due to the lower TRN. In this study, after the conversion, the NO_3_^−^ concentrations of purslane grown with 0, 100, 200, and 300 mM NaCl were 1868, 2081, 1,381, and 1,235 mg kg ^−1^ FW, respectively, and each of these values was much lower than 2,500 mg kg^−1^ FW as reported by Corrè and Breimer (1979). Purslane is a NO_3_^−^ accumulating plant; the exact contents depend on both cultivars (Egea-Gilabert et al., 2014) and growing conditions (Lara et al., 2011). Although our results showed that the leaf NO_3_^−^ concentrations of purslane grown hydroponically with different NaCl concentrations indoors under LED lighting were much lower than those reported by others, all plants had adequate shoot TRN which was greater than 2% (Figure 10B). According to Epstein (1999), an adequate tissue level of N that may be required by plants is around 1.5%. While reduced NO_3_^−^ concentration in the leaves of purslane contributes to good quality of this editable halophyte, concentrations of dietary minerals also contribute to nutritional profile (Uddin et al., 2012b). In the study of purslane, Chowdhar et al. (2013) reported that high salinity resulted in decreases of Ca, and K but increases of Mg and Fe concentration. Teixeira and Carvalho also reported that high salinity slightly increased Mg concentration. However, in this study, high salinity decreased dietary minerals such as K (Figure 12A), Ca (Figure 12B), and Mg (Figure 12C). Lower concentrations of K, Ca, and Mg in purslane grown with high salinity could be attributed to the stunted root architecture (Figure 2), which might have limited water and mineral uptake. Although statistically there were no significant differences in Fe among all treatments, salinity seems to increase Fe concentration (Figure 12D).

In conclusion, this study reveals that purslane is relatively tolerant to conditions of moderate salinity. It is feasible to grow purslane with 100 mM NaCl indoors hydroponically under LED lightings. Compared to those grown with fresh water (0 mM NaCl), purslane grown with 100 mM NaCl had greater shoot and root productivity and faster leaf growth and development and higher proline and Car concentrations. Increasing salinity from 100 mM to 200 mM and 300 mM resulted in decreases of shoot and root productions could be due to leaf water deficit reflected by lower LWC and reduced photosynthetic performance. Lower concentrations of Asc and total phenolic compounds of purslane grown with higher salinity in this study could mainly be due to its reduced photosynthetic performance. To enhance the nutritional quality of purslane plants without compromising its productivity, it would be feasible to first grow them with low salinity, such as 100 mM NaCl, to enhance photosynthetic performance, to achieve high biomass accumulation, and to increase mineral uptake before transferring to high salinity conditions. Increased shoot and root biomass accumulations and enhanced photosynthetic performance in purslane plants grown with low salinity may improve the production of phytochemicals after subjecting them to high salinity.

## Data Availability Statement

The raw data supporting the conclusions of this article will be made available by the authors, without undue reservation.

## Author Contributions

JH initiated and funded the expenses for the project and wrote the first draft of the manuscript. JH and LQ planned the experiments, carried out some parts of the experiments, and revised the manuscript. XY carried out most measurements, analyzed the data, and plotted the graphs under supervision of JH and LQ. All authors contributed to the article and approved the submitted version.

### Conflict of Interest

The authors declare that the research was conducted in the absence of any commercial or financial relationships that could be construed as a potential conflict of interest.

The handling editor declared a past co-authorship with one of the authors JH.
